# Interferon-*α* induces negative biases in emotional processing in patients with hepatitis C virus infection: a preliminary study

**DOI:** 10.1017/S0033291717002379

**Published:** 2017-09-11

**Authors:** C. M. Cooper, B. Godlewska, A. L. Sharpley, E. Barnes, P. J. Cowen, C. J. Harmer

**Affiliations:** 1 University Department of Psychiatry, Warneford Hospital Oxford, Oxford, UK; 2 NIHR Oxford Biomedical Research Centre, Oxford, UK; 3 The Peter Medawar Building for Pathogen Research, University of Oxford, Oxford, UK

**Keywords:** Depression, emotional processing, inflammation, interferon-*α*

## Abstract

**Background:**

Treatment of medical patients with the inflammatory cytokine, interferon-*α* (IFN-*α*), is frequently associated with the development of clinical depressive symptomatology. Several important biological correlates of the effect of IFN-*α* on mood have been described, but the neuropsychological changes associated with IFN-*α* treatment are largely unexplored. The aim of the present preliminary study was to assess the effect of IFN-*α* on measures of emotional processing.

**Method:**

We measured changes in emotional processing over 6–8 weeks in 17 patients receiving IFN-*α* as part of their treatment for hepatitis C virus infection. Emotional processing tasks included those which have previously been shown to be sensitive to the effects of depression and antidepressant treatment, namely facial expression recognition, emotional categorisation and the dot probe attentional task.

**Results:**

Following IFN-*α*, patients were more accurate at detecting facial expressions of disgust; they also showed diminished attentional vigilance to happy faces. IFN-*α* produced the expected increases in scores on depression rating scales, but there was no correlation between these scores and the changes in emotional processing.

**Conclusions:**

Our preliminary findings suggest that IFN-*α* treatment produces negative biases in emotional processing, and this effect is not simply a consequence of depression. It is possible that increased recognition of disgust may represent a neuropsychological marker of depressive disorders related to inflammation.

## Introduction

Interferon-*α* (IFN-*α*) is an endogenous cytokine that, when given as an exogenous therapy, potently activates the pro-inflammatory cytokine network to produce antiviral and antiproliferative effects (Capuron & Miller, 2004). Until recently, IFN-*α* was the most widely used treatment for hepatitis C virus (HCV) and was usually administered together with the oral polymerase inhibitor, ribavirin, for up to 48 weeks (Baraldi *et al.* 2012).

Despite its efficacy as an immunotherapy, IFN-*α* is strongly associated with the onset of neuropsychiatric symptoms, including anxiety, sleep alterations, fatigue and depressive symptomatology in 30–50% of patients (Musselman *et al.* 2001; Hauser *et al.* 2002; Schaefer *et al.* 2002). The clinical observation that IFN-*α* is capable of inducing symptoms that are strikingly similar to major depression is important evidence for the proposed role of endogenous pro-inflammatory cytokines in the development of depressive states more generally (Raison *et al.* 2006; Zunsain *et al.* 2013).

Several neurobiological mechanisms have been proposed as mediators of the ability of IFN-*α* to trigger depressive symptomatology. For example, it has been proposed that IFN-*α*-induced changes in tryptophan metabolism may lead to lowered brain serotonin levels and increased production of neurotoxic metabolites, such as quinolinic acid (Capuron *et al.* 2002; Miller, 2009). There is also preliminary evidence from magnetic resonance spectroscopy that IFN-*α*-induced alterations in glutamine and glutamate levels in the anterior cingulate cortex may correlate with depressive symptomatology (Haroon *et al.* 2014; Taylor *et al.* 2014). However, there has been little work to date on the neuropsychological mechanisms that may be involved in the mood-lowering effects of IFN-*α*.

Cognitive biases in emotional processing are thought to play a central role in the onset and maintenance of depression. For example, depression is associated with increased recognition, memory and attention to negative *v.* positive stimuli, thereby effectively exposing the patient to greater effects of any stressor, life event or negative social interaction. There is also robust evidence demonstrating that pharmacological modulation can modify emotional processing biases, as measured in psychological tasks, for example, following serotonergic antidepressant treatments (Harmer & Cowen, 2013). Importantly, during antidepressant treatment, changes in emotional processing occur prior to changes in subjective mood suggesting that they might be mediating mechanisms (Harmer *et al.* 2009*a*, *b*). A recent study also found that emotional processing bias in depression predicted subsequent changes in mood (Lewis *et al*. 2017). This study therefore aimed to investigate the effects of IFN-*α* administration on emotional processing, with a view to improving understanding of its action at a neuropsychological level and to assess whether deterioration in mood during IFN-*α* treatment is associated with changes in emotional processing. It was hypothesised that IFN-*α* treatment would induce negative biases in emotional processing reminiscent of a depressed state.

## Method

### Participants and study design

We recruited 21 participants who were scheduled to receive pegylated IFN-*α* plus ribavirin treatment as part of their routine clinical care for chronic HCV. Four participants did not return for the second study visit; therefore, data presented are from 17 participants (13 males, 4 females; mean age 39.7, s.d. 10.3, range 22–60 years). Participants were referred from a National Health Service hepatology clinic at the John Radcliffe Hospital (Oxford, UK). They were screened using the Structured Clinical Interview for DSM-IV (SCID; Spitzer *et al.* 1995) to be free of current Axis I mood and anxiety disorder; however, past psychiatric history of these disorders were not exclusion criteria. Participants taking non-stable psychotropic medication were excluded from the study, as were participants with clinical or biochemical evidence of cirrhosis. Concomitant medication was maintained at a stable dose throughout the study with the following medications being taken by participants: metformin and insulin (*n* = 1), ventolin inhaler (*n* = 1), lansoprazole (*n* = 1), omeprazole (*n* = 1), three patients were on stable doses of methadone and two patients were taking stable doses of the antidepressant mirtazapine. The study received full ethical approval by the Oxfordshire Research Ethics Committee A, and all participants gave written informed consent prior to study procedures being performed.

Participants attended two study visits; one pre-treatment with IFN and one after 6–8 weeks of IFN-*α* treatment. Mood state was assessed at baseline (pre-treatment) and 6–8 weeks after IFN-*α* administration using the Hamilton Depression Rating Scale (HAM-D; Hamilton, 1960), Beck Depression Inventory (BDI; Beck *et al.* 1961), state component of the State Trait Anxiety Inventory (Spielberger *et al*. 1970) and the Chalder Fatigue Scale (CFS; Chalder *et al.* 1993). Additionally, participants completed the above questionnaires (except the HAM-D) weekly for 6 weeks after the start of treatment to assess changes over time.

### Psychological tasks

#### Facial expression recognition

This task featured facial expressions of six basic emotions (happiness, surprise, sadness, fear, anger and disgust) taken from the Pictures of Affect Series (Ekman, 1976). Faces were morphed between each prototype and neutral to portray varying intensities of each emotion, in 10% steps from 0% (neutral) to 100% (full intensity of emotion). Four examples of each emotion at each intensity were displayed, in addition to 10 neutral stimuli, giving a total of 250 stimuli. Facial expressions were presented in random order for 500 ms, followed by a blank screen. Participants were asked to classify facial expressions as quickly and accurately as possible by pressing one of seven labelled keys on the keyboard. Accuracy (the number of correctly identified faces of a particular emotion divided by the total number of faces containing that emotion), reaction times for correct responses and misclassifications of emotional faces were calculated.

#### Emotional categorisation task

Sixty personality characteristic words selected to be disagreeable (e.g. domineering, hostile) or agreeable (e.g. optimistic, honest; taken from Anderson, 1968) were presented in a random order on screen for 500 ms. Words were matched in terms of length, ratings of frequency and meaningfulness, and equal numbers of positive and negative words were included. Participants were asked to imagine whether they would be pleased or upset if they were to overhear someone else describing them in this way, so that the judgement was self-referential in nature. Responses were made by pressing a labelled button according to whether they would ‘like’ or ‘dislike’ to be described as possessing that characteristic. Subjects were asked to respond as quickly and accurately as possible. Accuracy of categorisation and reaction times for correct identifications were calculated in this task.

#### Dot probe attentional task

Stimuli were pairs of facial expressions (Matsumoto & Ekman, 1988), each comprising one emotional and one neutral expression of the same individual or two neutral expressions of the same individual (described previously by Murphy *et al*. 2008). This meant that there were three types of face pairs: neutral–neutral, fearful–neutral and happy–neutral. Fearful and happy faces were presented with equal frequency. A central fixation cross began each trial, followed by pairs of faces, one at the top and one at the bottom of the screen. In the unmasked condition, each face pair was presented for 100 ms, immediately followed by a probe, which appeared in the location of one of the preceding facial expressions. The probe was two dots in a vertical (:) or horizontal (..) orientation. Participants were asked to identify the orientation of the dots by pressing the correspondingly labelled key on a keyboard. The probe remained on the screen until the participant had responded, and participants were asked to respond as quickly and accurately as possible. The masked condition consisted of the same sequence of events, except that the face pair was displayed for 16 ms followed by a mask (scrambled face), which was displayed for 84 ms. Each of the three face pair conditions were presented 32 times in the masked condition and 32 times in the unmasked condition, thus there were 192 trials in total. There were eight blocks of both the masked and unmasked conditions, which were presented in an alternating order. Position of an emotional face, probe position and type were fully counterbalanced. The task design therefore involved congruent trials (where the probe appears in the position of the emotional face) and incongruent trials (where the probe appears in the position of the neutral face while an emotional face is present).

Attentional vigilance scores were calculated for each participant by subtracting the median reaction time in congruent trials from incongruent trials. Positive values reflect attention towards the emotional face (vigilance), whereas negative values reflect attention away from the emotional face (avoidance).

### Statistical analysis

Statistical analyses were carried out using IBM SPSS Statistics (version 22). Questionnaire data [HAM-D, BDI, state anxiety and Chalder Fatigue Questionnaire (CFQ)] were compared before and after IFN-*α* treatment using paired sample *t* tests. To assess changes in subjective mood over the 6-week period after the start of treatment, repeated measures analyses of variance (ANOVAs) were performed with time as a within-subject factor. The facial expression recognition, emotional categorisation and dot probe tasks were all analysed using repeated measures ANOVA, with time (representing pre-treatment and treatment with IFN-*α*) and emotion as within-subject factors. In the dot probe task, masking was added as an additional within-subject factor. *Post hoc* analyses using paired sample *t* tests were performed to follow-up interactions observed. Where assumptions of equality of variances were broken, Greenhouse–Geisser procedure was used to correct the degrees of freedom. Extreme outlying data (data lying at more than three times the participants’ interquartile range above their third or below their first quartile) were removed from all psychological tasks. This resulted in minimal loss of data on any task (<2%). Data from each task were analysed separately, without correcting for multiple comparisons across tasks.

To assess the relationship between depressed mood and behavioural measures, Pearson's correlation coefficient was computed between depression severity scores (measured by the BDI) and neuropsychological task performance. Pearson's correlation coefficients were also examined between pre-treatment and post-treatment HAM-D and subjective mood scores, to assess whether pre-treatment mood was an indicator of depressed mood following IFN-*α* administration in this cohort.

## Results

### Clinical rating scales

IFN-*α* treatment significantly increased HAM-D scores [*t*(16) = −4.93, *p* < 0.001]. Furthermore, all subjective state measures of negative affect included in the study were significantly elevated after IFN-*α* treatment, as shown in Table 1. Mood, anxiety and fatigue scores recorded weekly for 6 weeks following the start of treatment increased in a linear manner with time; therefore, results presented here are from baseline and 6–8 weeks only.

**Table 1.** Mood state changes over time

	Baseline	IFN-*α*	Statistical significance
HAM-D	4.47 (5.75)	13.59 (9.18)	*p* < 0.001
BDI	6.32 (7.79)	14.56 (12.35)	*p* = 0.001
State anxiety	33.35 (8.29)	39.82 (17.27)	*p* = 0.045
CFQ	13.94 (5.54)	21.00 (7.14)	*p* < 0.001

HAM-D, Hamilton Depression Rating Scale; BDI, Beck Depression Inventory; CFQ, Chalder Fatigue Questionnaire.

Values are ratings at baseline (pre-treatment) and 6–8 weeks after IFN-*α* treatment (*n* = 17). Means (standard deviations).

Significant correlations between pre- and post-IFN-*α* depressed mood scores were found, suggesting that mood state at baseline predicted later depressed mood scores following IFN-*α* administration (HAM-D *r* = 0.56, *p* = 0.02; BDI *r* = 0.73, *p* = 0.001). State anxiety at baseline also correlated with elevated anxiety ratings following IFN-*α* therapy (*r* = 0.76, *p* < 0.001), although this was not significantly correlated with later depressed mood score (*p* > 0.1).

### Facial expression recognition

**Fig. 1.**
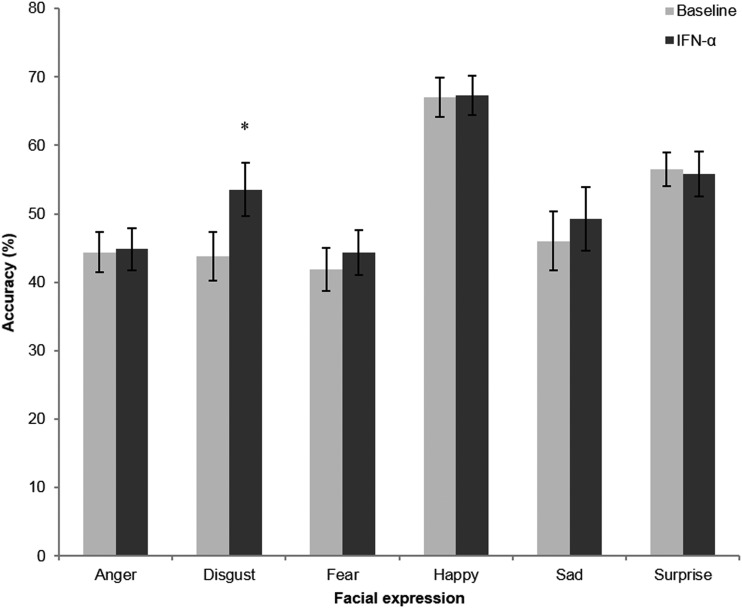
Performance in the facial expression recognition task before (light bars) and following 6–8 weeks IFN-*α* treatment. Values represent the mean percentage correct for each of the six basic emotions summed over the different intensity levels used in this task, with error bars representing standard error of the mean. *p < 0.001.

There were significant main effects of emotion [*F*(5,70) = 14.03, *p* < 0.001] and treatment [*F*(1,14) = 10.95, *p* = 0.005] on accuracy in this task, as well as a significant interaction between emotion and treatment [*F*(3.07,43.0) = 3.41, *p* = 0.025]. *Post hoc* analysis revealed that this was driven by significantly increased accurate recognition of disgust after IFN-*α* treatment [*t*(15) = −4.71, *p* < 0.001; see Fig. 1]. No significant effects were observed for other valences (anger *p* = 0.82, fear *p* = 0.25, happy *p* = 0.71, sad *p* = 0.38, surprise *p* = 0.77); hence, this appears to be a specific effect on disgust recognition.

For emotion discrimination (*d*′), which controls for differences in response criteria, the results were similar to those seen for accuracy above; there was a significant main effect of emotion [*F*(5,70) = 15.06, *p* < 0.001] and of treatment [*F*(1,14) = 12.16, *p* = 0.004]. In addition, there was a significant emotion x treatment interaction [*F*(3.06,42.81) = 3.05, *p* = 0.038]. Again, there was a marked increase for disgust sensitivity after IFN-*α* treatment compared with baseline; *post hoc* analysis revealed that this difference was highly significant [paired *t* test: *t*(15) = −5.20, *p* < 0.001]. There was a trend for increased sensitivity to fearful faces (*p* = 0.062), but no significant effects on other emotions (*p* > 0.2).

There were no significant correlations between depression rating and accuracy or reaction times in the facial expression recognition task either pre-treatment or following IFN-*α* administration (all *p* > 0.1).

### Emotional categorisation task

**Fig. 2.**
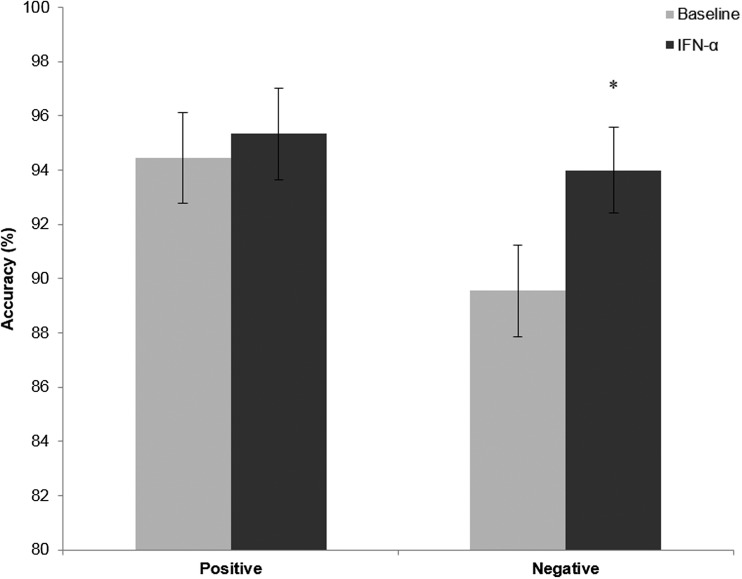
Accuracy in the emotional categorisation task before (light bars) and after IFN-*α* administration (dark bars). Values represent the mean percentage correct categorisation of positive and negative personality characteristic words used in this task, with error bars representing standard error of the mean. *p = 0.017.

There was a significant main effect of emotion [*F*(1,14) = 8.74, *p* = 0.01] and of treatment [*F*(1,14) = 9.6, *p* = 0.02] on accuracy in categorising self-referent personality characteristics, largely driven by increased accuracy in categorising negative characteristics [*t*(14) = −2.70, *p* = 0.017, Fig. 2]. However, there was no interaction between emotion and treatment [*F*(1,14) = 4.27, *p* = 0.24], so this should be interpreted with caution.

There were no treatment effects on reaction times to categorise self-referent personality characteristics in terms of a main effect of treatment [*F*(1,14) = 0.84, *p* = 0.38] or emotion x treatment interaction [*F*(1,14) = 2.04, *p* = 0.18], though there was a main effect of emotion [*F*(1,14) = 10.17, *p* = 0.007]. There were no correlations between depression score and accuracy or reaction times in the emotional categorisation task (all *p* > 0.1).

### Dot probe attentional vigilance

**Fig. 3.**
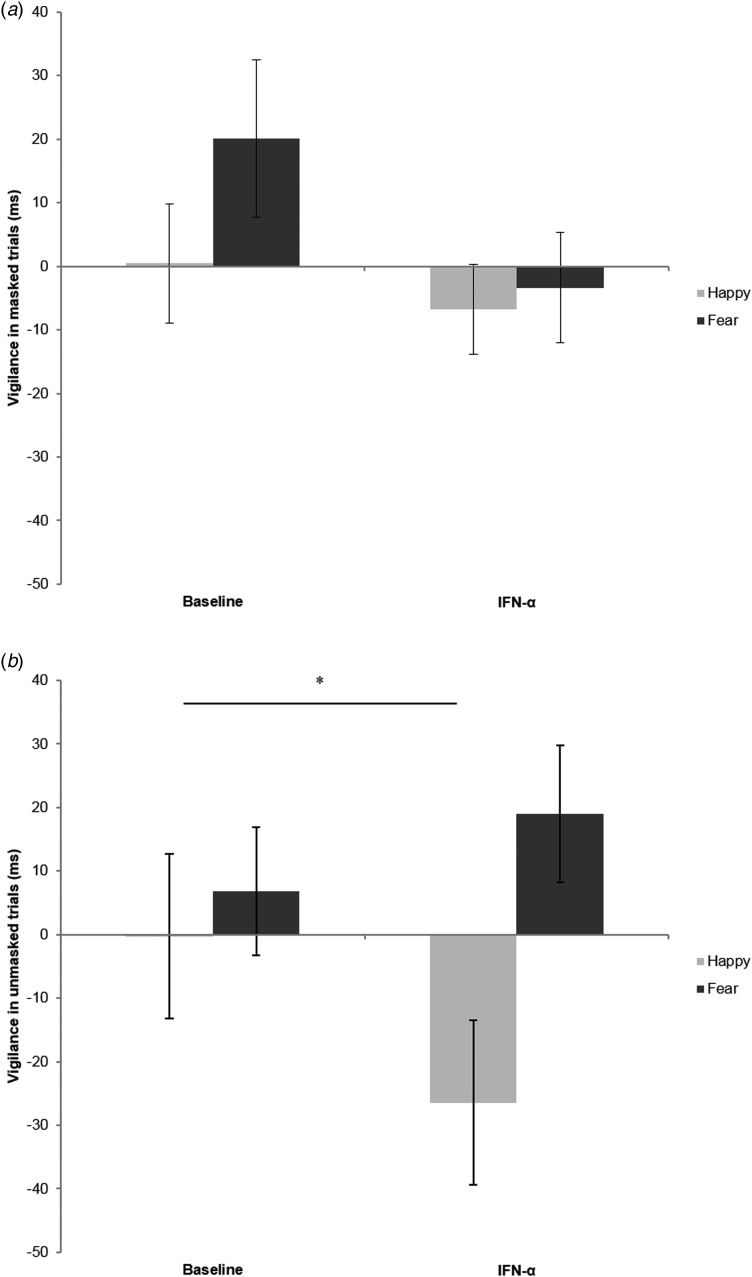
Effects of IFN-*α* on attentional vigilance for happy and fearful facial expressions in the masked condition (*a*) and unmasked condition (*b*) of the attentional dot probe task. Values are attentional scores before (baseline) and after IFN-*α* treatment. Attentional vigilance scores were calculated by subtracting the median reaction time from congruent trials (when the probe appeared in the same position as the emotional face) from incongruent trials (when the probe appeared in the opposite position to the emotional face, i.e. in the position of the neutral face). A positive score indicates vigilance towards the emotional face, whereas a negative score reflects avoidance of the emotional face. Error bars show the standard error of the mean. *p = 0.05.

There was a significant interaction between emotion, mask and treatment [*F*(1,16) = 8.32, *p* = 0.01], where mask was included as an additional within-subject factor (see Fig. 3). When masked and unmasked trials were considered separately, there was a significant emotion x treatment interaction in the unmasked [*F*(1,16) = 5.46, *p* = 0.03], but not masked [*F*(1,16) = 1.08, *p* = 0.31], condition. *Post hoc* analysis revealed that this was driven by a significant reduction in vigilance scores for happy faces in the unmasked condition following IFN-*α* treatment [*t*(16) = 2.10, *p* = 0.05]. There were no general effects of IFN-*α* on reaction times in this task (all *p* > 0.1).

### Correlations between psychological ratings and emotional processing tasks

Depressed mood scores measured by the BDI and HAM-D either at baseline or at 6 weeks were not significantly correlated with emotional processing task performance in the current study, for example, depressed mood was not associated with disgust recognition. There was also no significant correlation between change in depressed mood and change in disgust recognition (*p* > 0.4), emotional recognition (*p*>0.1) or dot probe performance (*p*⩾0.1). Finally, performance on the emotional processing tasks at baseline did not predict later depression scores at 6 weeks (*p*⩾0.1).

## Discussion

### Subjective ratings

IFN-*α* significantly increased depression severity ratings, as well as subjective ratings of state anxiety and fatigue by 6–8 weeks, which is in keeping with numerous previous reports of induction of depressive symptoms by this treatment (Musselman *et al.* 2001; Hauser *et al.* 2002; Schaefer *et al.* 2002; Capuron & Miller, 2004; Maddock *et al.* 2005; Fontana *et al.* 2008). Pre-treatment depressed mood scores were positively correlated with depressed mood following IFN-*α*, which also supports previous observations suggesting that severity of depressive symptoms during IFN-*α* treatment is predicted by baseline depression scores (Capuron & Ravaud, 1999; Musselman *et al.* 2001). However, neither depressed mood scores, nor change in scores over treatment measured by the BDI and HAM-D were significantly correlated with emotional processing task performance in the current study. This suggests that changes in emotional processing described here are not simply a consequence of change in mood.

Previous studies suggest that there is a time lag between cognitive bias and changes in symptoms of depression (Lewis *et al.* 2017), and therefore measuring mood and bias at the same time points may have obscured a mediating relationship, that is, that cognitive biases may affect mood over time via interaction with environmental inputs, such as life events, stressors and social interactions. As such we cannot rule out that early change in cognitive biases (e.g. after 1 week of treatment) may be associated with week 6 depression symptoms. It is also possible that the lack of association between change in cognitive bias and depression ratings at 6–8 weeks reflects a lack of power in our study.

### Facial expression recognition

The present preliminary study showed highly specific effects of IFN-*α* treatment on sensitivity to disgusted faces, including enhanced ability to discriminate disgusted faces, paired with reduced threshold of disgust detection. There were no effects of treatment on processing of other key emotions, and elevated disgust accuracy was not accompanied by enhancement of mislabelling other facial expressions as disgust.

Several previous studies have reported that increased disgust recognition might indicate a negative perceptual bias that is relevant to mood disorders, for example, elevated discrimination of disgusted facial expressions has been observed in euthymic medicated patients with bipolar disorder (Harmer *et al.* 2002). Hayward *et al*. (2005) also observed enhanced recognition of disgusted faces in a recovered depressed sample following tryptophan depletion. Similarly, tryptophan depletion in remitted depressed individuals speeded the recognition of disgust faces (Merens *et al.* 2008). In contrast, tryptophan supplementation, as opposed to depletion, reduced recognition of disgusted faces in healthy female volunteers (Murphy *et al.* 2006).

Despite considerable evidence of neural substrates involved in disgust processing and growing appreciation of disgust as an important feature in depression (Surguladze *et al.* 2010), interpretation of the role of disgust processing in depressive disorders lacks clarity. Emotional processing biases in disgust processing have been suggested to serve an evolutionary purpose of behavioural modification to avoid threats of infectious disease (Curtis *et al.* 2004), which could be related to the evidence that suggests that environmental and social disgusted stimuli are attended to and appraised for more time than fearful faces (Zhang *et al.* 2014). Enhanced disgust recognition may also be associated with visceral symptoms, such as nausea (Anderson *et al.* 2007). For example, increased recognition of disgusted faces was seen following duloxetine treatment, which induced nausea (Harmer *et al*. 2008). There are suggestions, however, that disgust processing can extend into the social domain, and that heightened disgust sensitivity might reflect an emotional processing bias that constitutes social and self-related disgust, feelings of social rejection (Rozin *et al.* 1994), as well as feelings of shame and guilt (Surguladze *et al.* 2010; Giner-Sorolla & Espinosa, 2011), all of which are highly relevant to depression. Emotional responses to inflammation are also relevant to evolutionary biological theories linking inflammation and depression in which depressive behaviours, regulated by the immune system, play a role in dealing with infection both at the individual level and that of the social group (Anders *et al*. 2013).

It should be noted that although discussion here has focussed on enhanced disgust sensitivity, some studies report no difference in disgust processing in depression (Bediou *et al.* 2005), while others have reported impaired disgust recognition (Douglas & Porter, 2010). When consideration is given to the heterogeneity of symptomatology in depression, in addition to differing compensatory adaptations, perhaps this variability in disgust processing is not surprising.

Such divergent evidence makes it difficult to reliably conclude the meaning of enhanced disgust recognition following immune system activation in the current study. It seems conceivable, however, that effects on disgust recognition observed could represent a complex interplay between inflammatory pathways and neurocircuits that result in modified information processing of aversive disgusted facial emotions, which may in part be modulated by serotonin function (Anderson *et al.* 2007). Indeed, recent fMRI neuroimaging studies support a role for the insula in the effects of inflammatory challenge (Harrison *et al*. 2016), and the insula is a key node in processing cues of disgust (Surguladze *et al.* 2010). A previous study also reported increased activity within subgenual anterior cingulate cortex and reduced connectivity to amygdala, medial prefrontal cortex and the nucleus accumbens during emotional face processing following an inflammatory challenge (Harrison *et al*. 2016). Thus, the current pattern may represent a cognitive correlate of the underlying neurocircuits emerging as important for the effects of inflammation in depression (Byrne *et al.* 2016).

### Emotional categorisation

The increased accurate categorisation of negative self-referent personality words observed following IFN-*α* therapy is suggestive of a negative bias in emotional processing, though it should be interpreted with caution given the lack of a statistically significant interaction with valence in the full ANOVA. Negative biases in emotional categorisation have previously been reported in depressed patients, reversal of which has been associated with later antidepressant response. For example, slower reaction times to positive trait words were found in depressed patients compared with healthy controls; however, after a single dose of reboxetine, reaction times to positive adjectives were faster (Harmer *et al.* 2009*b*). Further studies have reported similar antidepressant effects on emotional categorisation with reboxetine (Harmer *et al.* 2004), citalopram (Harmer *et al.* 2004) and mirtazapine (Arnone *et al.* 2009).

### Attentional dot probe

The current findings in the attentional dot probe task suggest that administration of IFN-*α* produces negative biases in attentional vigilance, particularly by increasing attentional avoidance of happy facial expressions in the unmasked condition. Negative biases in emotional processing include both vigilance towards negative stimuli and away from positive stimuli (Disner *et al.* 2011). Depressed patients have previously been shown to display an attentional bias away from positive (happy) faces and towards negative (sad) faces (Gotlib *et al.* 2004; Fritzsche *et al.* 2010) and negative words (Bradley *et al.* 1997).

It has been previously proposed that in the neutral–happy face pair presented in this task, the neutral face signifies the more threatening facial stimuli when compared with the happy face. Therefore, avoidance of the happy face in the current study indicates attention towards the neutral face, which may be relevant to threat-related processing biases (Cooper & Langton, 2006). This would also be in keeping with the (non-significant) observation of enhanced vigilance to threatening fearful faces with IFN-*α* treatment.

Tianeptine, an antidepressant drug with suggested properties of reducing serotonin function early in treatment was also shown to significantly enhance avoidance to happy faces (Cooper *et al.* 2015) in a very similar manner to effects seen here with IFN-*α*. Tryptophan supplementation, in contrast, has been shown to reduce vigilance to negative words in healthy female volunteers (Murphy *et al.* 2006). It is too soon to conclude the way in which IFN-*α* may be exerting this effect, though it is intriguing to note that once again, as in the increased disgust recognition after IFN-*α*, the literature would be consistent with a reduction in serotonin availability as a possible mechanism.

### Limitations

There are some important limitations in the present study. Firstly, a sample size of 17 participants is relatively small, particularly given known variance in mood and behavioural response to IFN-*α* treatment as not all patients become depressed. The sample size is also limiting since it does not allow reliable stratification of those patients who did exhibit depressive symptoms, compared with those who did not. This would be helpful to investigate whether baseline neuropsychological task performance is able to predict mood response to treatment, and thereby identify those at elevated risk of developing IFN-*α*-associated depression. Further limitations of this study include the lack of control subjects with HCV receiving placebo and not IFN-*α* treatment, which would have helped to examine cause and effect in relation to depression symptoms, as well as learning effects on task performance. However, previous work examining the test–retest effects in this same battery have shown limited learning effects of repeat testing in the absence of any intervention (Thomas *et al.* 2016). Future work should nonetheless examine the specificity of the negative bias induction seen here relative to placebo treatment in this patient group.

The lack of accurate accounts of somatic symptoms on testing days could have also been useful with regards to accounting for visceral symptoms, such as nausea (Anderson *et al.* 2007), which can unfortunately be a side effect of IFN-*α* treatment and, as discussed above, may be involved in altered disgust recognition. It is also possible that the changes in emotional processing we have identified are secondary to the mood changed produced by IFN-*α*. Against this, we could not find any correlation between depression scores and emotional processing.

The present study does not have any biological markers, including inflammatory cytokines or tryptophan and its metabolites, which could have provided insight into the biological mechanisms associated with behavioural effects observed. Furthermore, mediation analysis factoring in inflammatory cytokines measures could have facilitated assessment of the mediator of psychological effects observed and potentially informed the lack of correlation between mood scores and behavioural findings.

## Conclusions

The present study provides intriguing evidence that IFN-*α*, either directly or perhaps indirectly via pro-inflammatory cytokine pathways, is capable of producing negative emotional processing biases that are widely thought to be important in the onset and maintenance of depression. As such, these results highlight that exposure to inflammation by immune system activation may alter affective information processing, and that this could play a role in the development of depression over time in subgroups of patients. While there are many similarities between the present findings and the depression literature, there are some interesting differences, particularly the highly specific enhancement of disgust recognition. Although this has previously been seen in mood disorders, it could represent an effect that is more specific to inflammatory pathways.

## References

[ref1] Anders S, Tanaka M, Kinney DK (2013). Depression as an evolutionary strategy for defense against infection. Brain, Behavior, and Immunity 31, 9–22. 10.1016/j.bbi.2012.12.002 23261774

[ref2] Anderson IM, Del-Ben CM, McKie S, Richardson P, Williams SR, Elliott R, Deakin JFW (2007). Citalopram modulation of neuronal responses to aversive face emotions: a functional MRI study. Neuroreport 18, 1351–1355. 17762711 10.1097/WNR.0b013e3282742115

[ref3] Anderson NH (1968). Likableness ratings of 555 personality-trait words. Journal of Personality and Social Psychology 9, 272–279. 5666976 10.1037/h0025907

[ref4] Arnone D, Horder J, Cowen PJ, Harmer CJ (2009). Early effects of mirtazapine on emotional processing. Psychopharmacology 203, 685–691. 19031070 10.1007/s00213-008-1410-6

[ref5] Baraldi S, Hepgul N, Mondelli V, Pariante CM (2012). Symptomatic treatment of interferon-alpha-induced depression in hepatitis C: a systematic review. Journal of Clinical Psychopharmacology 32, 531–543. 22722514 10.1097/JCP.0b013e31825d9982

[ref6] Beck AT, Ward CH, Mendelson M, Mock J, Erbaugh J (1961). An inventory for measuring depression. Archives of General Psychiatry 4, 561–571. 13688369 10.1001/archpsyc.1961.01710120031004

[ref7] Bediou B, Krolak-Salmon P, Saoud M, Henaff MA, Burt M, Dalery J (2005). Facial expression and sex recognition in schizophrenia and depression. Canadian Journal of Psychiatry 50, 525–533. 16262107 10.1177/070674370505000905

[ref8] Bradley BP, Mogg K, Millar N, Bonham-Carter C, Fergusson E, Jenkins J (1997). Attentional biases for emotional faces. Cognition and Emotion 11, 25–42.

[ref9] Byrne M, Whittle S, Allen N (2016). The role of brain structure and function in the association between inflammation and depressive symptoms: a systematic review. Psychosomatic Medicine 78, 389–400. 26910795 10.1097/PSY.0000000000000311

[ref10] Capuron L, Miller AH (2004). Cytokines and psychopathology: lessons from interferon-alpha. Biological Psychiatry 56, 819–824. 15576057 10.1016/j.biopsych.2004.02.009

[ref11] Capuron L, Ravaud A (1999). Prediction of the depressive effects of interferon alfa therapy by the patient's initial affective state. New England Journal of Medicine 340, 1370. 10223879 10.1056/NEJM199904293401716

[ref12] Capuron L, Ravaud A, Neveu PJ, Miller AH, Maes M, Dantzer R (2002). Association between decreased serum tryptophan concentrations and depressive symptoms in cancer patients undergoing cytokine therapy. Molecular Psychiatry 7, 468–473. 12082564 10.1038/sj.mp.4000995

[ref13] Chalder T, Berelowitz G, Pawlikowska T, Watts L, Wessely S, Wright D (1993). Development of a fatigue scale. Journal of Psychosomatic Research 37, 147–153. 8463991 10.1016/0022-3999(93)90081-p

[ref14] Cooper CM, Whiting DA, Cowen PJ, Harmer CJ (2015). Tianeptine in an experimental medicine model of antidepressant action. Journal of Psychopharmacology 29, 582–590. 25759404 10.1177/0269881115573810

[ref15] Cooper RM, Langton SR (2006). Attentional bias to angry faces using the dot-probe task? It depends when you look for it. Behaviour Research and Therapy 44, 1321–1329. 16321361 10.1016/j.brat.2005.10.004

[ref16] Curtis V, Aunger R, Rabie T (2004). Evidence that disgust evolved to protect from risk of disease. Proceedings of the Royal Society B: Biological Sciences 271(Suppl 4), S131–S133. 15252963 10.1098/rsbl.2003.0144 PMC1810028

[ref17] Disner SG, Beevers CG, Haigh EA, Beck AT (2011). Neural mechanisms of the cognitive model of depression. Nature Reviews Neurosciences 12, 467–477. 10.1038/nrn3027 21731066

[ref18] Douglas KM, Porter RJ (2010). Recognition of disgusted facial expressions in severe depression. British Journal of Psychiatry 197, 156–157. 20679270 10.1192/bjp.bp.110.078113

[ref19] Ekman PF (1976). Pictures of Facial Affect. Consulting Psychologists Press: Palo Alto, California.

[ref20] Fontana RJ, Kronfol Z, Lindsay KL, Bieliauskas LA, Padmanabhan L, Back-Madruga C (2008). Changes in mood states and biomarkers during peginterferon and ribavirin treatment of chronic hepatitis C. American Journal of Gastroenterology 103, 2766–2775. 18721241 10.1111/j.1572-0241.2008.02106.x PMC3712502

[ref21] Fritzsche A, Dahme B, Gotlib IH, Joormann J, Magnussen H (2010). Specificity of cognitive biases in patients with current depression and remitted depression and in patients with asthma. Psychological Medicine 40, 815–826. 19719897 10.1017/S0033291709990948 PMC2847035

[ref22] Giner-Sorolla R, Espinosa P (2011). Social cuing of guilt by anger and of shame by disgust. Psychological Science 22, 49–53. 21156860 10.1177/0956797610392925

[ref23] Gotlib IH, Krasnoperova E, Yue DN, Joormann J (2004). Attentional biases for negative interpersonal stimuli in clinical depression. Journal of Abnormal Psychology 113, 121–135. 14992665 10.1037/0021-843X.113.1.121

[ref24] Hamilton M (1960). A rating scale for depression. Journal of Neurology Neurosurgery and Psychiatry 23, 56–62. 10.1136/jnnp.23.1.56 PMC495331 14399272

[ref25] Harmer CJ, Cowen PJ (2013). ‘It's the way that you look at it’ – a cognitive neuropsychological account of SSRI action in depression. Proceedings of the Royal Society B: Biological Sciences 368, 20120407. 10.1098/rstb.2012.0407 PMC3638386 23440467

[ref26] Harmer CJ, Goodwin GM, Cowen PJ (2009a). Why do antidepressants take so long to work? A cognitive neuropsychological model of antidepressant drug action. British Journal of Psychiatry 195, 102–108. 19648538 10.1192/bjp.bp.108.051193

[ref27] Harmer CJ, Grayson L, Goodwin GM (2002). Enhanced recognition of disgust in bipolar illness. Biological Psychiatry 51, 298–304. 11958780 10.1016/s0006-3223(01)01249-5

[ref28] Harmer CJ, Heinzen J, O'Sullivan U, Ayres RA, Cowen PJ (2008). Dissociable effects of acute antidepressant drug administration on subjective and emotional processing measures in healthy volunteers. Psychopharmacology 199, 495–502. 18575851 10.1007/s00213-007-1058-7

[ref29] Harmer CJ, O'Sullivan U, Favaron E, Massey-Chase R, Ayres R, Reinecke A (2009b). Effect of acute antidepressant administration on negative affective bias in depressed patients. American Journal of Psychiatry 166, 1178–1184. 19755572 10.1176/appi.ajp.2009.09020149

[ref30] Harmer CJ, Shelley NC, Cowen PJ, Goodwin GM (2004). Increased positive versus negative affective perception and memory in healthy volunteers following selective serotonin and norepinephrine reuptake inhibition. American Journal of Psychiatry 161, 1256–1263. 15229059 10.1176/appi.ajp.161.7.1256

[ref31] Haroon E, Woolwine BJ, Chen X, Pace TW, Parekh S, Spivey JR (2014). IFN-alpha-induced cortical and subcortical glutamate changes assessed by magnetic resonance spectroscopy. Neuropsychopharmacology 39, 1777–1785. 24481242 10.1038/npp.2014.25 PMC4023151

[ref32] Harrison NA, Voon V, Cercignani M, Cooper EA, Pessiglipone M, Critchley HD (2016). A neurocomputational account of how inflammation enhances sensitivity to punishments versus rewards. Biological Psychiatry 80, 73–81. 26359113 10.1016/j.biopsych.2015.07.018 PMC4918729

[ref33] Hauser P, Khosla J, Aurora H, Laurin J, Kling MA, Hill J (2002). A prospective study of the incidence and open-label treatment of interferon-induced major depressive disorder in patients with hepatitis C. Molecular Psychiatry 7, 942–947. 12399946 10.1038/sj.mp.4001119

[ref34] Hayward G, Goodwin GM, Cowen PJ, Harmer CJ (2005). Low-dose tryptophan depletion in recovered depressed patients induces changes in cognitive processing without depressive symptoms. Biological Psychiatry 57, 517–524. 15737667 10.1016/j.biopsych.2004.11.016

[ref35] Lewis G, Kounali D-Z, Button KS, Duffy L, Wiles NJ, Munafo MR, Harmer CJ, Lewis G (2017). Variation in the recall of socially relevant information and depressive symptom severity: a prospective cohort study. Acta Psychiatrica Scanda 135, 489–498. 10.1111/acps.12729 PMC5763395 28374430

[ref36] Maddock C, Landau S, Barry K, Maulayah P, Hotopf M, Cleare AJ (2005). Psychopathological symptoms during interferon-alpha and ribavirin treatment: effects on virologic response. Molecular Psychiatry 10, 332–333. 15655564 10.1038/sj.mp.4001634

[ref37] Matsumoto D, Ekman P (1988). Japanese and Caucasian Facial Expressions of Emotion *(*JACFEE*)*. Intercultural and Emotion Research Laboratory, Department of Psychology, San Francisco State University: San Francisco, CA.

[ref38] Merens W, Booij L, Haffmans PJ, van der Does A (2008). The effects of experimentally lowered serotonin function on emotional information processing and memory in remitted depressed patients. Journal of Psychopharmacology 22, 653–662. 18308809 10.1177/0269881107081531

[ref39] Miller AH (2009). Mechanisms of cytokine-induced behavioral changes: psychoneuroimmunology at the translational interface. Brain, Behavior and Immunity 23, 149–158. 10.1016/j.bbi.2008.08.006 PMC2745948 18793712

[ref40] Murphy SE, Downham C, Cowen PJ, Harmer CJ (2008). Direct effects of diazepam on emotional processing in healthy volunteers. Psychopharmacology 199, 503–513. 18581100 10.1007/s00213-008-1082-2 PMC2493525

[ref41] Murphy SE, Longhitano C, Ayres RE, Cowen PJ, Harmer CJ (2006). Tryptophan supplementation induces a positive bias in the processing of emotional material in healthy female volunteers. Psychopharmacology 187, 121–130. 16767422 10.1007/s00213-006-0401-8

[ref42] Musselman DL, Lawson DH, Gumnick JF, Manatunga AK, Penna S, Goodkin RS (2001). Paroxetine for the prevention of depression induced by high-dose interferon alfa. New England Journal of Medicine 344, 961–966. 11274622 10.1056/NEJM200103293441303

[ref43] Raison CL, Capuron L, Miller AH (2006). Cytokines sing the blues: inflammation and the pathogenesis of depression. Trends in Immunology 27, 24–31. 16316783 10.1016/j.it.2005.11.006 PMC3392963

[ref44] Rozin P, Lowery L, Ebert R (1994). Varieties of disgust faces and the structure of disgust. Journal of Personality and Social Psychology 66, 870–881. 8014832 10.1037//0022-3514.66.5.870

[ref45] Schaefer M, Engelbrecht MA, Gut O, Fiebich BL, Bauer J, Schmidt F (2002). Interferon alpha (IFNalpha) and psychiatric syndromes: a review. Progress in Neuropsychopharmacology and Biological Psychiatry 26, 731–746. 10.1016/s0278-5846(01)00324-4 12188106

[ref46] Spielberger CD, Gorsuch RL, Lushene RD (1970). STAI Manual. Consulting Psychologists Press: Palo Alto, Calif.

[ref47] Spitzer RL, Williams JBW, Gibbon M, First MB (1995). Structured Clinical Interview for DSM-IV *(*SCID*)*. New York State Psychiatric Institute, Biometrics Research: New York.

[ref48] Surguladze SA, El-Hage W, Dalgleish T, Radua J, Gohier B, Phillips ML (2010). Depression is associated with increased sensitivity to signals of disgust: a functional magnetic resonance imaging study. Journal of Psychiatric Research 44, 894–902. 20307892 10.1016/j.jpsychires.2010.02.010 PMC4282743

[ref49] Taylor MJ, Godlewska B, Near J, Christmas D, Potokar J, Collier J (2014). Effect of interferon-*α* on cortical glutamate in patients with hepatitis C: a proton magnetic resonance spectroscopy study. Psychological Medicine 44, 789–795. 23659574 10.1017/S0033291713001062 PMC3758755

[ref50] Thomas JM, Higgs S, Dourish CT (2016). Test–retest reliability and effects of repeated testing and satiety on performance of an emotional test battery. Journal of Clinical and Experimental Neuropsychology 38, 416–433. 26702993 10.1080/13803395.2015.1121969 PMC4784484

[ref51] Zhang D, Liu Y, Wang X, Chen Y, Luo Y (2014). The duration of disgusted and fearful faces is judged longer and shorter than that of neutral faces: the attention-related time distortions as revealed by behavioral and electrophysiological measurements. Frontiers in Behavioral Neuroscience 8, 293. 25221488 10.3389/fnbeh.2014.00293 PMC4145716

[ref52] Zunsain PA, Hepgul N, Pariante CM (2013). Inflammation and depression. Current Topics in Behavioral Neurosciences 14, 135–151. 22553073 10.1007/7854_2012_211

